# A phospha-oseltamivir–biotin conjugate as a strong and selective adhesive for the influenza virus

**DOI:** 10.1016/j.bmcl.2014.02.021

**Published:** 2014-04-01

**Authors:** Hansjörg Streicher, Stephen R. Martin, Peter J. Coombs, John McCauley, David Neill-Hall, Mathew Stanley

**Affiliations:** a 18, Tarret Burn, Didcot OX11 7FZ, UK; b Chemistry Division, School of Life Sciences, University of Sussex, Brighton BN1 9QJ, UK; c Division of Physical Biochemistry, MRC National Institute of Medical Research, Mill Hill, London NW7 1AA, UK; d Division of Virology, MRC National Institute of Medical Research, Mill Hill, London NW7 1AA, UK

**Keywords:** Influenza virus, Oseltamivir, Neuraminidase inhibitor, Biosensors, Virus immobilisation

## Abstract

We present the synthesis and application of a molecule containing both the powerful influenza neuraminidase (NA) inhibitor phospha-oseltamivir and d-biotin, connected via an undecaethylene glycol spacer. It inhibits influenza virus neuraminidase (from the H3N2 X31 virus) in the same range as oseltamivir, with a slow off-rate, and produces a stable NA-coated surface when loaded onto streptavidin-coated biosensors. Purified X31 virus binds to these loaded biosensors with an apparent dissociation constant in the low picomolar range and binding of antibodies to the immobilized virus could be readily detected. The compound is thus a potential candidate for the selective immobilization of influenza virus in influenza diagnosis, vaccine choice, development or testing.

## Graphical abstract

*K*_d_ = 11 pM for influenza virus X31-binding to biosensors coated with **1**.

Selective and effective detection, immobilization and characterization of influenza viruses is an essential part of a large variety of tests and experiments in influenza diagnosis and influenza vaccine production.^1^ Immobilization of viruses can be achieved in either an unspecific manner, for instance direct immobilization on polystyrene plates, or by specific binding to either immobilized anti-influenza antibodies or to immobilized carbohydrates or glycoproteins (e.g. fetuin) that carry terminal sialic acid residues. The latter interaction occurs mainly through the influenza virus hemagglutinin (HA) and necessitates a high degree of multivalency (many carbohydrates interacting with many HA’s). Antibody cross-reactivity testing (essential in influenza vaccine selection and characterization) is currently severely dependent on the standard hemagglutination inhibition assay (HI). The HI assay involves examining the amount of antibody (serum) needed to inhibit the non-specific cross-linking of red blood cells by influenza virus (red blood cell cross-linking occurs mostly via the virus HA binding to sialic acid bearing carbohydrates on the red blood cell surface). All these methods, though widely used, have their individual shortcomings.^2,3^ Carbohydrate based immobilization strategies suffer from leaching effects and variations in affinities of the influenza viruses for particular carbohydrates. Antibody immobilization can suffer similar problems, as well as competition in binding when examining binding of antibodies/serum (a primary use of virus immobilization). There is also a major problem with current H3N2 virus strains (which continues to circulate and predominates in many regions of the world) in that they have notoriously weak binding to carbohydrate ligands via HA,^3–5^ meaning standard assays (such as the HI assay) are becoming more difficult to perform and difficult to interpret, thus representing an ongoing problem for reference centres and vaccine producers.

Immobilization of influenza viruses via their surface neuraminidase (NA) is much less established despite influenza NA inhibitors such as oseltamivir, zanamivir or peramivir displaying a much stronger monovalent interaction with NA than sialic acids binding to HA.^6,7^ We have designed phospha-oseltamivir–biotin conjugate **1**, containing an undecaethylene glycol spacer moiety, which inhibits NA (from H3N2 X31 virus)^8^ in the subnanomolar range (*K*_i_ = 1.8 nM), similar to conjugate **2** containing only a short spacer (*K*_i_ = 0.24 nM) and only slightly weaker than oseltamivir itself (*K*_i_ = 0.12 nM). Compound **2**, reported by us earlier,^9^ failed to immobilize virus effectively when tested with streptavidin-coated biosensors, most likely because it is unable to bind to virus and streptavidin simultaneously (not published). This indicated the importance of spacer type and length for the envisaged immobilization (Fig. 1).

*Synthesis*: In brief, azido derivative **3** is reduced to the amine with trimethyl phosphine and then coupled with *O*-[2-(Biotinylamino)ethyl]-*O*′-(2-carboxyethyl)undecaethylene glycol using PyBOP (Scheme 1). The resulting protected target molecule **4** was then deprotected in two steps using previously published procedures to give **1**. in high yield.^9,10^

*Neuraminidase inhibition*: In the well-established MUNANA assay,^11^ compound **1** tested with purified N2 neuraminidase (from the H3N2 X31 virus) gave *K*_i_ = 1.8 ± 0.4 nM (*k*_on_ = 5.6 × 10^4^ M^−^^1^ s^−^^1^, *k*_off_ = 1 × 10^−^^4^ s^−^^1^ (estimated from *K*_i_ × *k*_on_)). This compares with oseltamivir, *K*_i_ = 0.12 ± 0.3 nM (*k*_on_ = 2 × 10^6^ M^−^^1^ s^−^^1^, *k*_off_ = 2.4 × 10^−^^4^ s^−^^1^ (estimated from *K*_i_ × *k*_on_)). Hence compound **1** binds more slowly and dissociates a little more slowly than oseltamivir, and inhibits 15-fold more weakly, but still in the low nanomolar range. The high affinity and slow off-rate of compound **1** binding by neuraminidase means that it is highly suitable for immobilization of virus particles on streptavidin-coated surfaces.

*Surface interaction analysis*: Binding of neuraminidase and virus to compound **1** was measured on an Octet RED biolayer interferometer (Pall ForteBio Corp., Menlo Park, CA, USA). The compound was stably immobilized on streptavidin biosensors (Pall ForteBio Corp., Menlo Park, CA, USA) at a concentration of 0.5 μg/ml. All measurements were performed in 10 mM HEPES (pH 7.4), 150 mM NaCl, 3 mM EDTA and 0.005% Tween-20 at 25 °C.Binding of NA (0.1–20 nM) was measured at 25 °C with a 50 min association step. Binding of virus (1–400 pM) was measured with a 200 min association step. In both cases the response at the end of the association step was used to determine the equilibrium dissociation constant.

Purified N2 neuraminidase (from X31) bound to immobilised compound **1**, with an equilibrium dissociation constant, *K*_d_ = 3.4 nM (see Fig. 2, upper graph), which compares well with the *K*_i_ determined in the MUNANA assay (despite standard buffers and temperatures being somewhat different in the different experiments). No binding was observed for compound **2** due to the shorter linker.

Figure 2 (lower graph) shows that purified X31 virus^12^ binds to compound **1** coated biosensors, with an apparent equilibrium dissociation constant, *K*_d_ of 11 pM, a 300-fold increase in affinity compared to the binding of the purified neuraminidase due to multivalent avidity effects from binding of multiple neuraminidases on the surface of the virus simultaneously (Fig. 2).

*Virus immobilization and antibody binding*: To show the potential of the immobilization strategy using streptavidin-coated biosensors as described above, we have demonstrated that the binding of several different antibodies^13^ to immobilized X31 can be detected (see Fig. 3). The method therefore has the potential to be used in examining the cross-reactivity of antibodies/sera between different virus strains, an essential part of influenza vaccine choice, development and testing.

In conclusion, we have presented a new compound suitable to effectively immobilize the influenza virus on streptavidin-coated surfaces based on strong binding to the influenza NA. We believe it has good chance to be used by laboratories charged with the task of finding new solutions to the ongoing threat of influenza epidemics and pandemics.

## Figures and Tables

**Figure 1:**
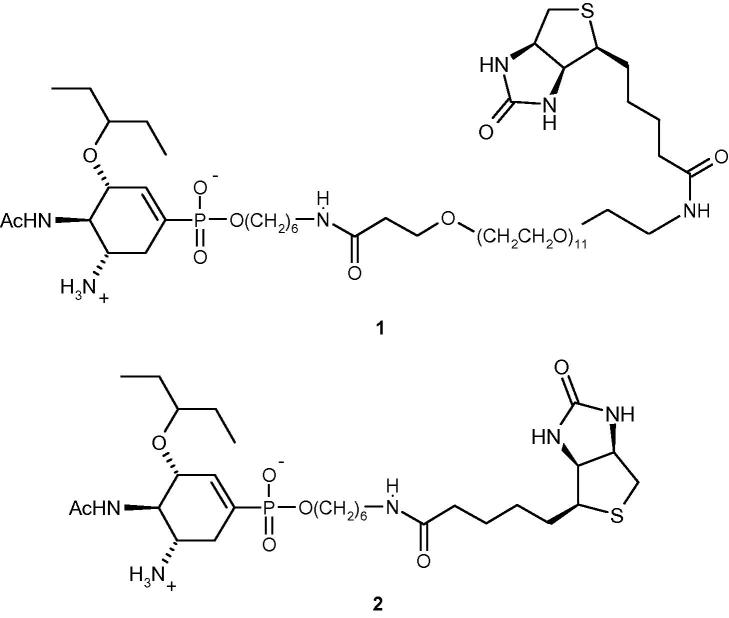
Phospha-oseltamivir conjugated to d-biotin with (**1**) and without undecaethyleneglycol spacer (**2**).

**Figure 2:**
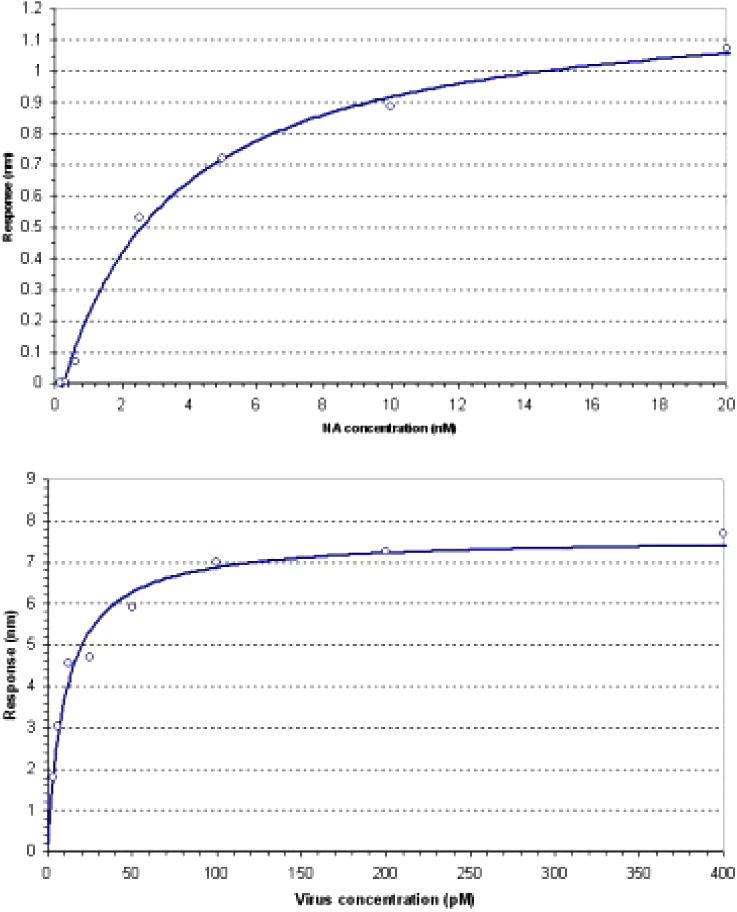
NA binding (upper graph) and virus X31 binding (lower graph) to streptavidin-coated biosensors loaded with compound **1**.

**Figure 3:**
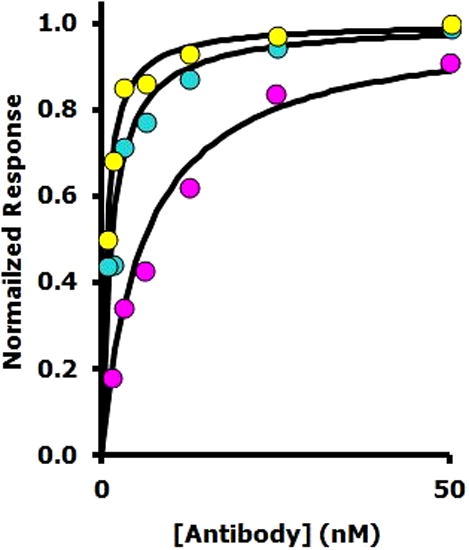
Binding of monoclonal antibodies Hc73 (yellow, *K*_d_ ∼ 0.7 nM), Hc100 (green, *K*_d_ ∼ 1.5 nM) and Hc221 (purple, *K*_d_ ∼ 6.5 nM) to immobilized X31.

**Scheme 1:**
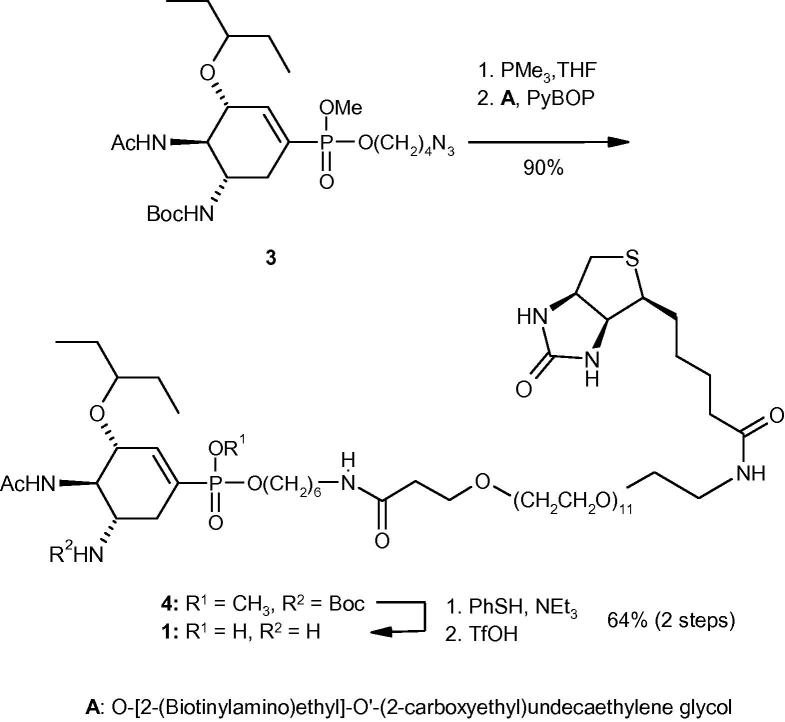
Synthesis of conjugate **1**.

## Supplementary data

Supplementary data Synthetic procedures and analytical data for compounds **1** and **4**.
