# Supplementary material for: A phospha-oseltamivir–biotin conjugate as a strong and selective adhesive for the influenza virus
Source: Bioorg Med Chem Lett. 2014 Apr 1;24(7):1805–7. doi: 10.1016/j.bmcl.2014.02.021 (PMC3988921; doi:10.1016/j.bmcl.2014.02.021)

Syntheses and analyses of conjugates 1 and 4

General

Where anhydrous solvents were required for reactions, MeOH and DMF were purchased (anhydrous) and used as received. DCM was doubly distilled (over CaH_2) before use and THF was obtained anhydrous and was used without further drying. All other solvents were dried using appropriate drying reagents. Fine chemicals were purchased from Aldrich-, Sigma- or Acros-Chemicals and were of the highest purity available. Reactions were monitored via thin layer chromatography (TLC) using pre-coated silica sheets with fluorescent indicator UV_{254}. Compound detection was achieved by UV absorption and by developing plates by staining with a molybdenum phosphate reagent (20 g ammonium molybdate and 0.4 g cerium$^{(IV)}$ sulfate in 400 mL of 10% aqueous sulphuric acid) with subsequent heating.

Chromatographic purification was performed using silica gel 60A ‘Davisil’ (particle size 35-70µm) from Fisher Scientific, UK and silica gel 100 C18 reversed phase (particle size 40-63µm from Fluka Analytical, UK. Silica-based MPLC chromatography was carried out on the Büchi Sepacore system equipped with glass columns packed with LiChroprep Si 60 (15-25µm) from Merck, Darmstadt, Germany. Solvents for chromatography were used as received except for toluene and ethyl acetate, which were distilled before use. Gel permeation chromatography was carried out in the 1-10 mg scale on a XK 16/70 column (bed volume 130 mL), from Amersham packed with Sephadex G-10 (particle size 40-120µm) and 0.1 M NH_4HCO_3 as buffer. Detection was achieved using a differential refractometer from Knauer, Berlin, Germany.

^{1}H NMR, ^{13}C NMR, ^{31}P NMR and all multidimensional spectra were recorded on Varian VNMRS spectrometers (600 MHz, 500 MHz or 400 MHz). Chemical shifts in ^{1}H and ^{13}C NMR spectra were referenced to the residual proton resonance of the respective deuterated solvents, $CDCl_3$ (7.26 ppm), D_2O (4.80 ppm) and CD_3OD (3.31 ppm) respectively. For ^{31}P NMR spectra H_3PO_4 was used as external standard (0 ppm).

HR-ESI-MS spectra were recorded on a Bruker Daltonics Apex III in positive mode with MeOH and/or H_2O as solvent. Where possible, HR-ESI-MS has been used to characterise compounds which have been synthesised.

Target compound phospha-oseltamivir-biotin conjugate 1

Under an atmosphere of dry nitrogen, protected biotin-conjugate **4** (26 mg, 0.019 mmol) was dissolved in THF (1.5 mL), NEt_3 (0.37 mL, 0.27 mmol) and thiophenol (0.016 mL, 0.133 mmol) were added and the mixture was stirred for 48 h at room temperature. The same amounts of NEt_3 and thiophenol were added and strirring was continued for another 24 h when tlc indicated completion of the reaction. The solvents and reagents were removed *in vacuo* and the resulting crude product was taken up in water (2 mL), sonicated and filtered. The demethylated intermediate was purified by gel

permeation chromatography (0.1 M NH_4HCO_3) as detailed in ref. 6, and the resulting 13 mg (51%) were stirred in a solution of TFA/H_2O (1:1) (1 mL) overnight. The solvent was then evaporated *in vacuo* and the residue was purified again by gel permeation chromatography (0.1 M NH_4HCO_3) to afford target compound **1** (8 mg, 66%).

^{1}H NMR (600 MHz, D_2O) δ_H: 0.89 – 1.00 (6 H, m, $-OCH(CH_2C\underline{H_3})_2$), 1.42 – 1.76 (18 H, bm, $-OCH(C\underline{H_2}CH_3)_2$, $-C(O)CH_2\text{-}C\underline{H_2}\text{-}C\underline{H_2}\text{-}C\underline{H_2}\text{-}$, $-OCH_2C\underline{H_2}C\underline{H_2}C\underline{H_2}C\underline{H_2}CH_2NH\text{-}$), 2.13 (3 H, s, $-NHCOC\underline{H_3}$), 2.27, (2 H, m, $-C(O)\underline{CH_2}\text{-}$), 2.48, 2.59 (2 H, m, H_{6ax}, H_{6eq}), 2.85, 3.02 (2 H, d, dd, J = 13.1 Hz, J = 5.0, 13.2 Hz, $-S\text{-}\underline{CH_2}\text{-}$), 3.25 (1H, $OC\underline{H}(CH_2CH_3)_2$, 3.42-3.45 (3 H, $-CH_2C\underline{H_2}NH\text{-}$, $-S\text{-}\underline{CH}\text{-}$), 3.66 – 3.72 (4 H, m, $-OCH_2\underline{CH_2}NHCO\text{-}$, $NHCO\underline{CH_2}CH_2O\text{-}$), 3.74-3.77 (44H, $-O(\underline{CH_2CH_2})_{11}O\text{-}$), 3.79-3.90 (6H, m, $-O\underline{CH_2}CH_2NHCO\text{-}$, $-NHCOCH_2\underline{CH_2}O\text{-}$, H_4, H_5), 3.98 (1H, m, H_3), 4.27 (2 H, m, $-OC\underline{H_2}CH_2\text{-}$), 4.49 (1 H, m, $-S\text{-}CH\text{-}C\underline{H}\text{-}NHCO\text{-}$), 4.67 (1 H, $-S\text{-}CH_2\text{-}C\underline{H}\text{-}NHCO\text{-}$), 6.37 (1 H, d, $J_{P\text{-}2}$ = 19.06 Hz, H_2).

^{13}C NMR (150.8 MHz, D_2O) δ_C: 8.93, 9.01 ($-OCH(CH_2\underline{C}H_3)_2$), 22.72 ($-NHCO\underline{C}H_3$), 25.12, 25.52, 25.59, 25.94, 26.12, 28.07, 28.25, 28.59, (3 biotin-C, $-OCH_2CH_2\underline{C}H_2\underline{C}H_2\text{-}\underline{C}H_2CH_2NH\text{-}$, $-OCH(\underline{C}H_2CH_3)_2$), 30.26 ($-OCH_2\underline{C}H_2\text{-}$), 35.83 (C-biotin), 36.51 (C_6), 39.63 (2 C-biotin), 49.6 (d, C5), 53.52 (C4), 55.73, 60.62, 62.45 (3C-biotin), 65.68, 67.23, 68.31, 69.26, 69.65, 69.79, 69.88, 70.03 ($-O\underline{C}H_2\underline{C}H_2O\text{-}$, $O\underline{C}H_2CH_2NHCO\text{-}$, $PO\underline{C}H_2\text{-}$, $-NHCOCH_2\underline{C}H_2\text{-}$), 76.28 (C3), 84.51 ($-O\underline{C}H(CH_2CH_3)_2$), 130.0 ($C_1$), 136.45 ($C_2$), 166.68 ($-NH\text{-}\underline{C}(O)\text{-}NH\text{-}$), 175.12 ($-NH\underline{C}OCH_3$), 175.53 ($-CH_2\text{-}\underline{C}(O)NH\text{-}CH_2\text{-}$), 177.25 ($-NH\underline{C}OCH_2CH_2$).

^{31}P NMR (242.7 MHz, MeOH-D_4) δP: 13.13 (s).

HR-ESI-MS (m/z) calculated for $C_{56}H_{105}N_6O_{20}PS$ $[M+H]^+$ 1246.65, found 1246.6960.

Protected phospha-oseltamivir-biotin conjugate 4

Under an atmosphere of dry nitrogen, azide **3**[6] (29 mg, 0.052 mmol) was was dissolved in dry THF (1 mL) and PMe_3 (1 M stock solution in THF, 68 µL) was added dropwise with stirring. When tlc indicated the absence of starting material (~ 4h), the reaction was quenched with deionised water (0.5 mL) and the mixture was stirred for an additional 30 minutes. The solvent was then removed *in vacuo* and the crude product was placed on a short silica plug (DCM/MeOH; 10:1 +3% NEt_3) to give the respective amine without additional purification. d-Biotin (51 mg, 0.060 mmol) and PyBOP (32 mg, 0.062 mmol) were dried *in vacuo* and then placed under an N_2 (g) atmosphere. Dry DMF (1 mL) was then added followed by DIPEA (16 µL, 0.0974 mmol). The reaction flask was then sonicated briefly and cooled to 0 °C (ice-bath). After a few minutes the isolated amino-conjugate (23 mg, 0.0487 mmol) was dissolved in dry DMF (1 mL) and was added dropwise to the stirring d-Biotin solution. The solution was allowed to warm slowly to room temperature overnight after which time TLC indicated the reaction was complete. The solvent was evaporated *in vacuo* and then purified by flash

chromatography (EA/MeOH; 1:2 + 2% AcOH) to give protected target compound **4** (46 mg, 56%). R_f : 0.4 (EA/MeOH; 1:2 + 2% AcOH).

^{1}H NMR (500 MHz, MeOH-d_4) δ_H: 0.84, 0.96 (6 H, 2t, J = 7.4 Hz, -OCH($CH_2C\underline{H_3}$)$_2$), 1.43 (9 H, s, -NHCOC($C\underline{H_3}$)$_3$), 1.34 – 1.78 (18 H, bm, -OCH($C\underline{H_2}CH_3$)$_2$, -C(O)CH_2-$C\underline{H_2}$-$C\underline{H_2}$-$C\underline{H_2}$-, -$OCH_2C\underline{H_2}$-$C\underline{H_2}C\underline{H_2}$-$C\underline{H_2}CH_2$NH-), 1.92 (3 H, s, -NHCO$C\underline{H_3}$), 2.24 (2 H, dd, J = 6.4 Hz, -C(O)$\underline{CH_2}$-), 2.24 (1 H, m, H_{6ax}), 2.51 (1 H, m, H_{6eq}), 2.72, 2.93 (2 H, d, dd, J = 12.7, 4.7, 12.6 Hz, -S-$\underline{CH_2}$-), 3.16-3.24 (3 H, m, -$CH_2C\underline{H_2}$NH-, -S-$\underline{CH}$-), 3.41 (1 H, m, -OC$\underline{H}$(CH_2CH_3)$_2$), 3.55 (4H, m, -$OCH_2\underline{CH_2}$NHCO-, -NHCO$\underline{CH_2}CH_2$O-), 3.63-3.66 (m, 47H, -O$\underline{CH_2}\underline{CH_2}$O-, P(O)O$\underline{CH_3}$), 3.72 – 3.74 (5 H, m, -O$\underline{CH_2}CH_2$NHCO-,-NHCO$CH_2\underline{CH_2}$O-, H_5), 3.86 (1 H, m, H_4), 4.00 – 4.14 (3 H, m, -OC$\underline{H_2}CH_2$-, H_3), 4.31 (1 H, dd, J = 4.3, 7.4 Hz, -S-CH-C$\underline{H}$-NHCO-), 4.51 (1 H, dd, J = 5.8, 6.0 Hz, -S-CH_2-C$\underline{H}$-NHCO-), 6.57 (1 H, d, J_{P-2} = 22.0 Hz, H_2).

^{13}C NMR (151 MHz, MeOH-d_4) δ_C: 9.71, 10.02 (-OCH($CH_2\underline{C}H_3$)$_2$), 23.05 (-NHCO$\underline{C}H_3$), 26.43, 26.86, 26.93, 29.60, 29.86, 30.37 (C(O)CH_2-$\underline{C}H_2$-$\underline{C}H_2$-$\underline{C}H_2$-, -OCH_2CH_2-$\underline{C}H_2\underline{C}H_2$-$\underline{C}H_2CH_2$NH-), 27.36, 27.53 (-OCH($\underline{C}H_2CH_3$)$_2$), 28.81 (-NHCOC($\underline{C}H_3$)$_2$), ~31.4 (d, J = 5.9 Hz, -$OCH_2\underline{C}H_2$-), 32.1, 36.86, 40.23 (-$CH_2\underline{C}H_2$NH-, biotin-C, C_6), 41.07 (biotin-C,), 50.4 (C5), 53.4 (d, J = 5.8 Hz, -P(OC$\underline{H_3}$)$_2$), 56.60 (m, C4), 57.08, 61.70, 63.45 (biotin-C), 67.84, 68.29, 70.68, 71.39, 71.41, 71.51, 71.56, 71.62, 71.64 (-O$\underline{C}H_2\underline{C}H_2$O-, (-O$\underline{C}H_2CH_2$NH-, -PO$\underline{C}H_2$-, -NHCO$CH_2\underline{C}H_2$-), 77.4 (C3), 80.39 (-NHCO$\underline{C}$(CH_3)$_2$), 83.89 (-O$\underline{C}$H(CH_2CH_3)$_2$), 144.32 (C2), 157.97 (-NH$\underline{C}$OC(CH_3)$_2$), 166.15 (-NH-$\underline{C}$(O)-NH-), 173.88 (-NH$\underline{C}$OCH_3), 176.18 (-CH_2-$\underline{C}$(O)NH-CH_2-).

^{31}P NMR (161.7 MHz, MeOH-d_4) δP: 19.00 (s).

HR-ESI-MS (m/z) calculated for $C_{62}H_{115}N_6O_{22}PS$ $[M+Na]^+$ 1381.75, found 1381.7471.

NMR-Spectra

1H-NMR Spectrum Compound 1

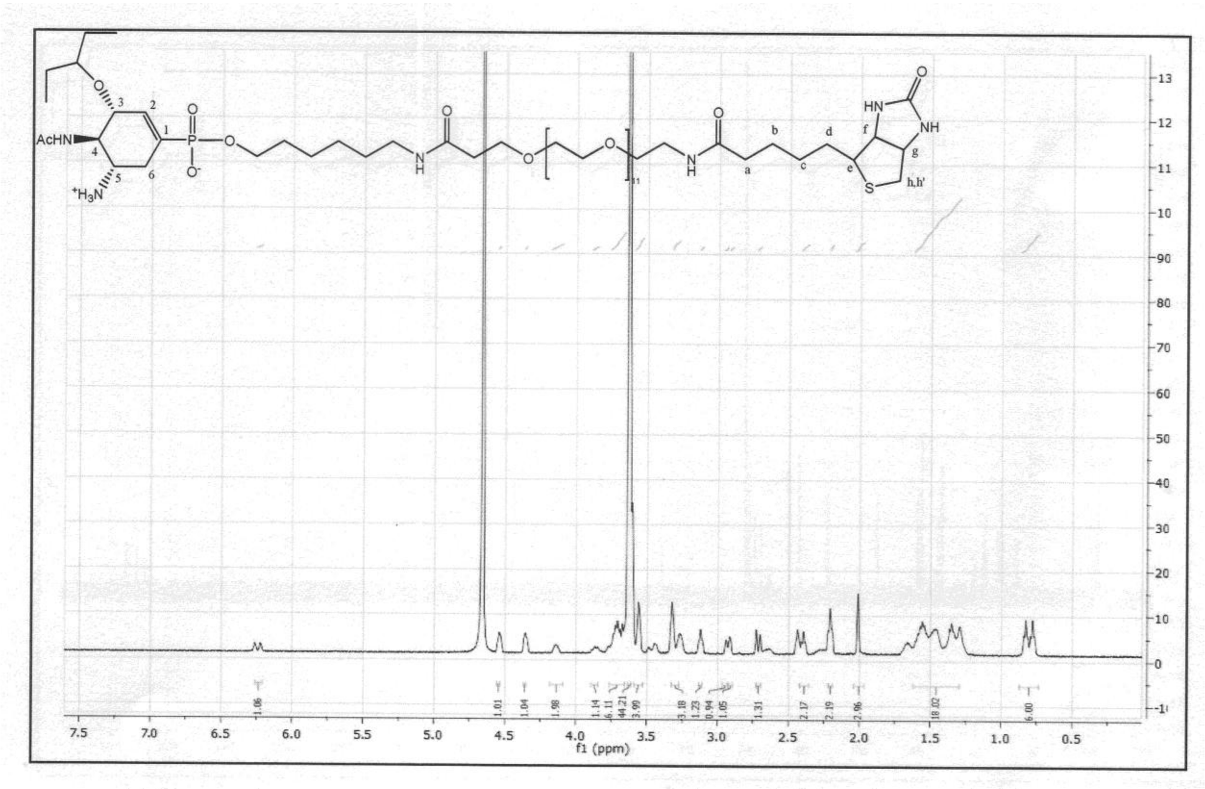

13C-NMR Spectrum Compound 1

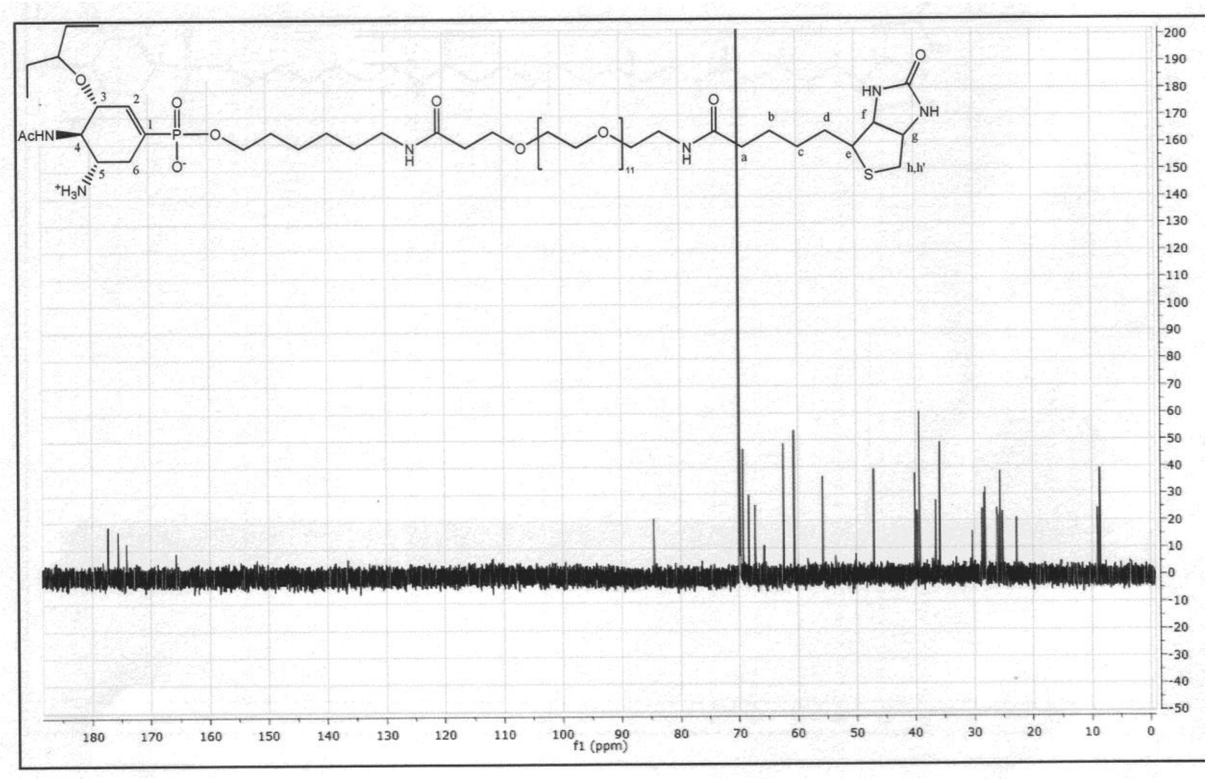

31P-NMR Spectrum Compound 1

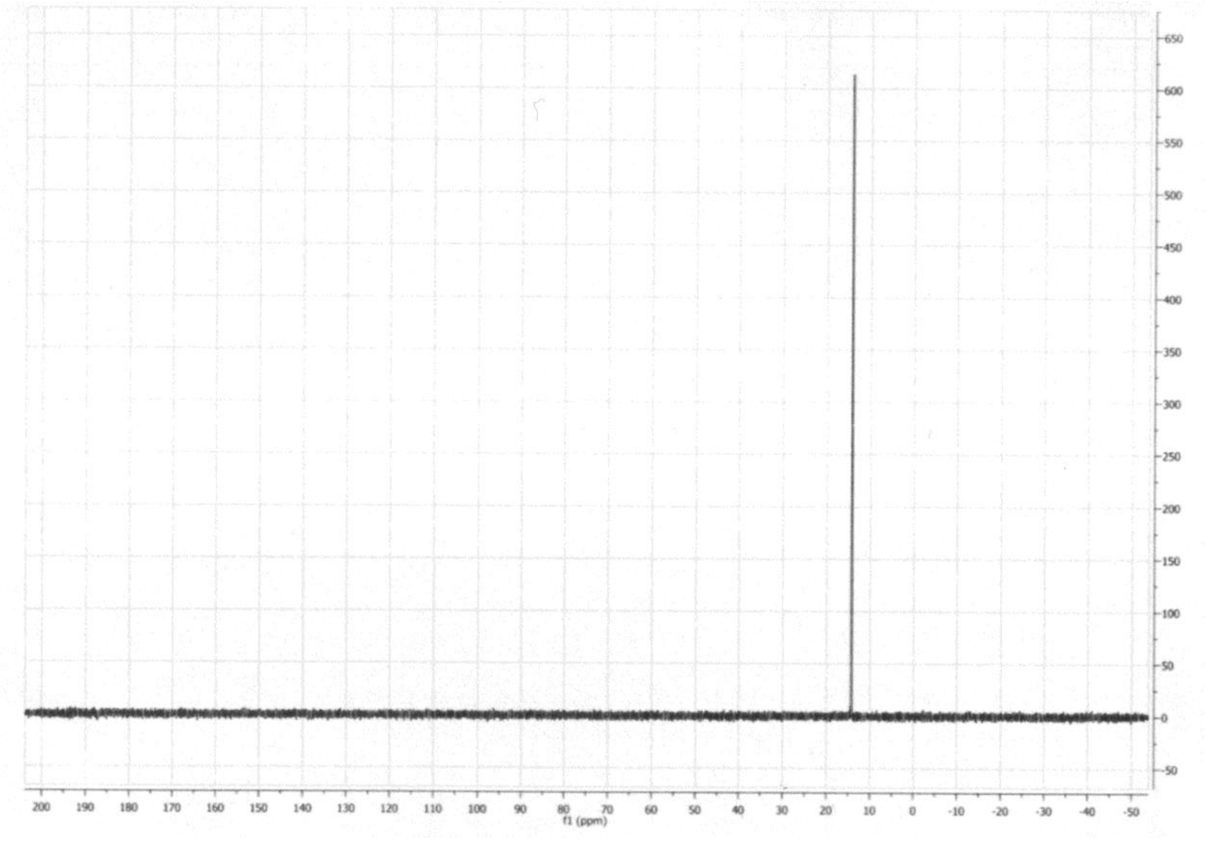

1H-NMR Spectrum Compound 4

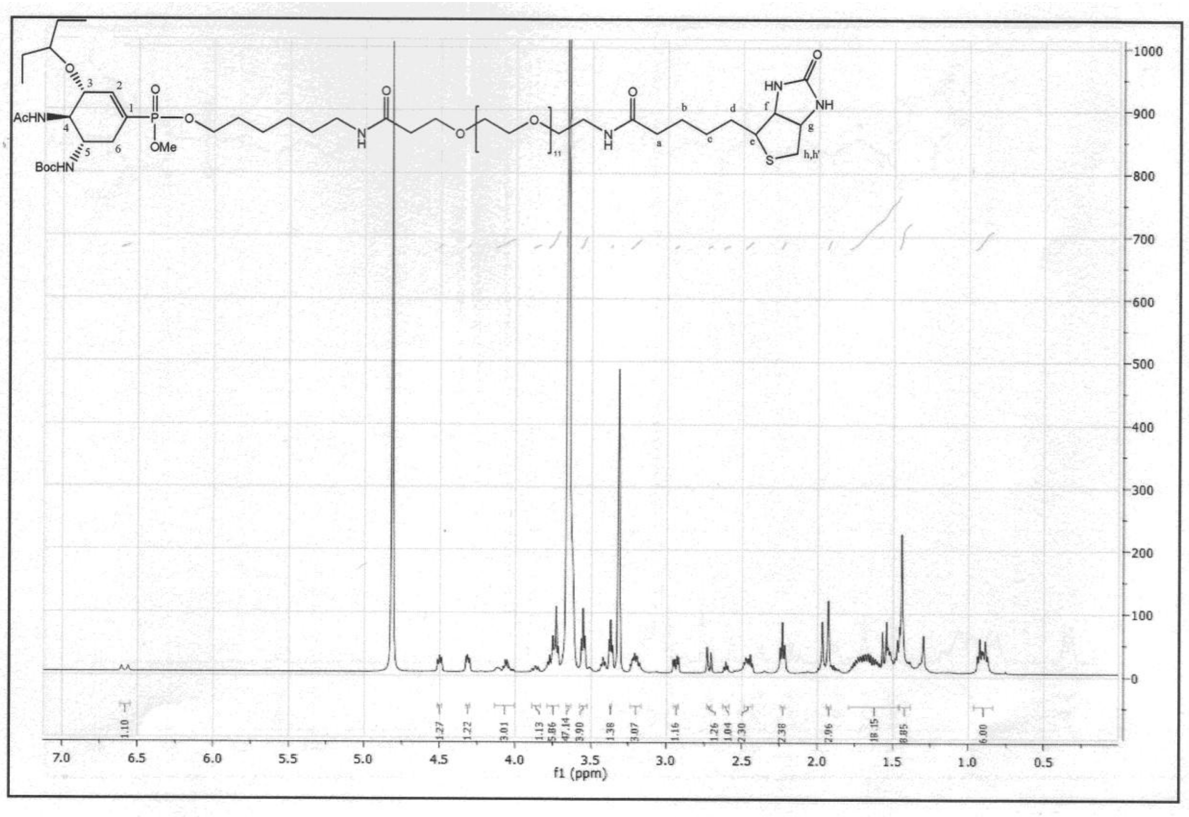

13C-NMR Spectrum Compound 4

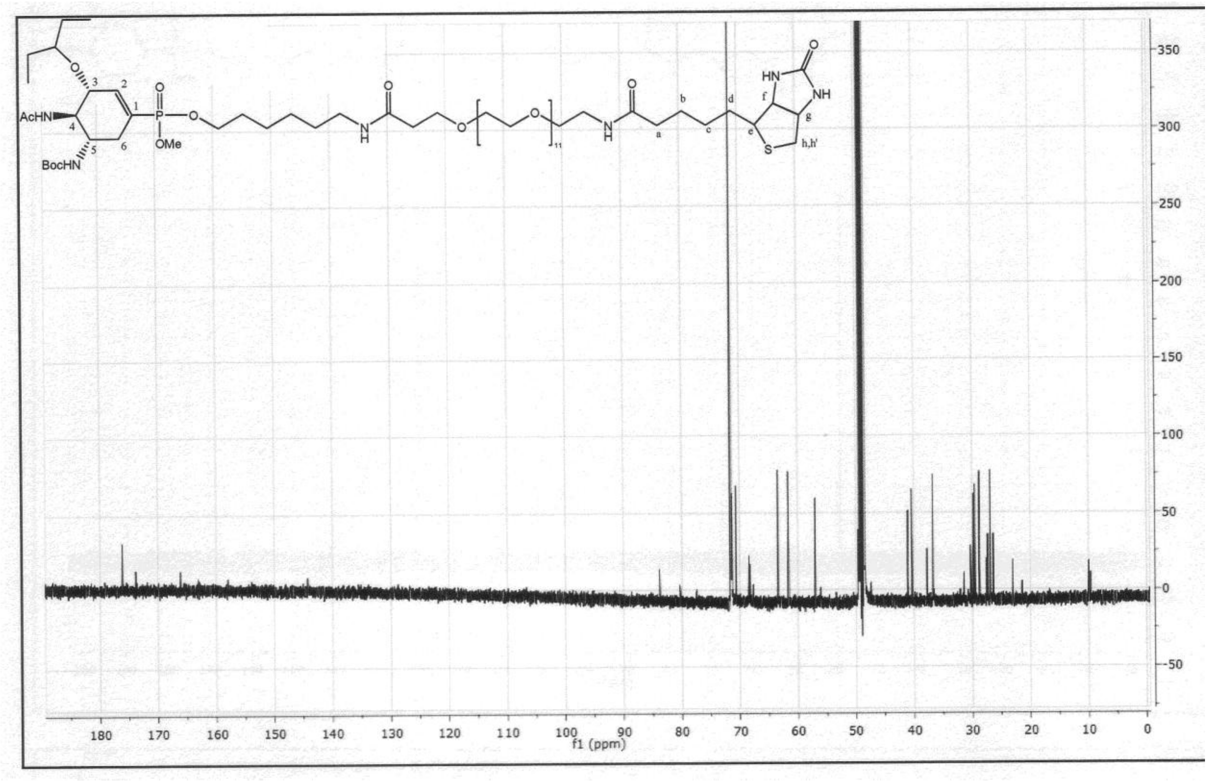

31P-NMR Spectrum Compound 4

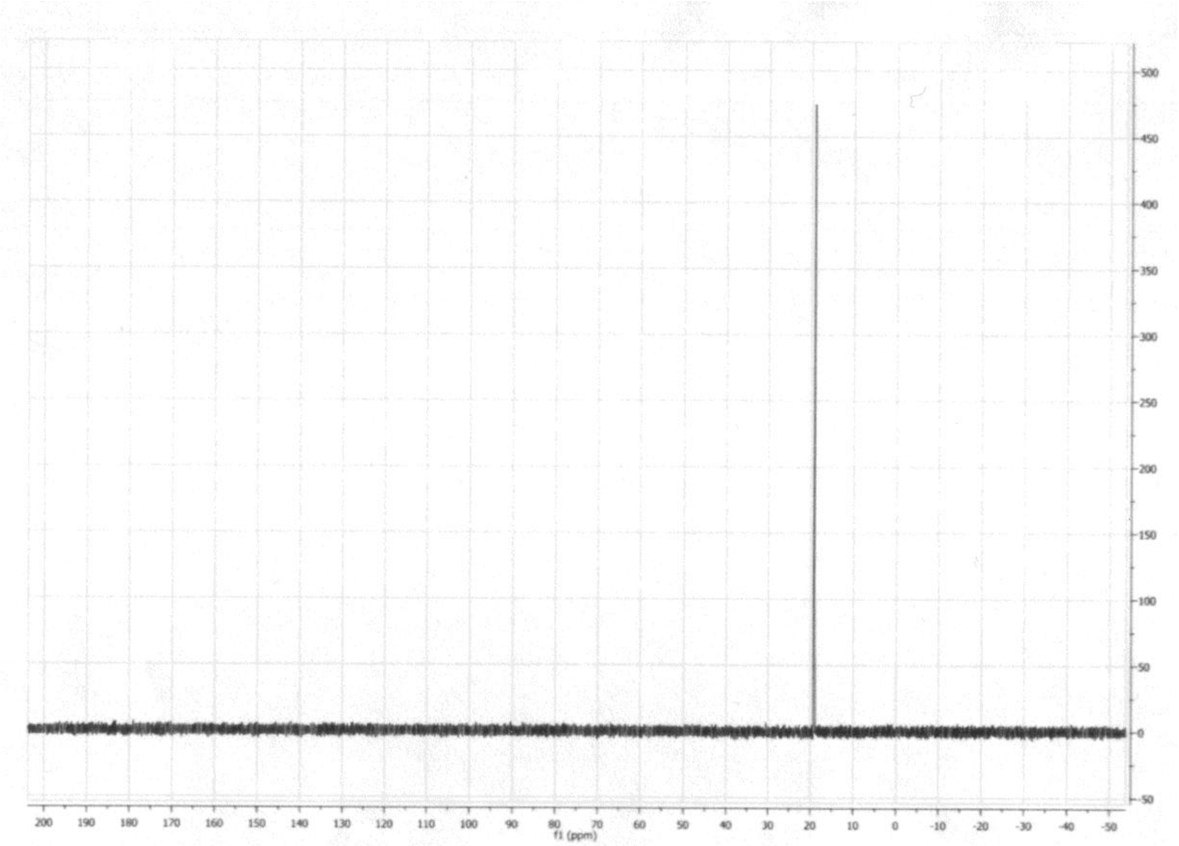

Supplement: Supplementary data — Synthetic procedures and analytical data for compounds 1 and 4. [file mmc1.pdf]
